# A Comparative Analysis of the Definitions of Autonomous Weapons Systems

**DOI:** 10.1007/s11948-022-00392-3

**Published:** 2022-08-23

**Authors:** Mariarosaria Taddeo, Alexander Blanchard

**Affiliations:** 1 Oxford Internet Institute, University of Oxford, Oxford, UK; 2 Alan Turing Institute, London, UK

**Keywords:** Adapting capabilities, Autonomy, Autonomous artificial agents, Autonomous weapons systems, Artificial intelligence, Definition, Human control, Lethal autonomous weapons systems.

## Abstract

In this report we focus on the definition of autonomous weapons systems (AWS). We provide a comparative analysis of existing official definitions of AWS as provided by States and international organisations, like ICRC and NATO. The analysis highlights that the definitions draw focus on different aspects of AWS and hence lead to different approaches to address the ethical and legal problems of these weapons systems. This approach is detrimental both in terms of fostering an understanding of AWS and in facilitating agreement around conditions of deployment and regulations of their use and, indeed, whether AWS are to be used at all. We draw from the comparative analysis to identify essential aspects of AWS and then offer a definition that provides a value-neutral ground to address the relevant ethical and legal problems. In particular, we identify four key aspects—autonomy; adapting capabilities of AWS; human control; and purpose of use—as the essential factors to define AWS and which are key when considering the related ethical and legal implications.

## Introduction

The debate on the ethical and legal implications of autonomous weapons systems (AWS) dates back to the early 2000s, with some proponents (Arkin, 2009) defending the use of these systems and others calling for a ban (Sharkey, 2008, 2010; Sparrow, 2007). The debate has become much more active since 2012, when the US Department of Defence (DoD) published an executive order on AWS (US Department of Defense, 2012) which, along with the report from Human Rights Watch (‘Losing Humanity: The Case against Killer Robots’ 2012), revamped the international debate on the ethical and legal problems posed by AWS. Since then, the debate has grown with contributions from scholars, military and policy experts, and the involvement of the International Committee of the Red Cross (ICRC), the UN Institute for Disarmament Research (UNIDIR), and the UN Convention on Certain Weapons (CCW), which established a Governmental Group of Experts (GGE) to discuss emerging technologies in the area of lethal autonomous weapon systems (LAWS).

While the debate remains deeply polarised as to whether the use of AWS is ethically acceptable and legally sound, there is at least consensus as to what ethical and legal aspects are to be considered in making this call: respect of human dignity, International Humanitarian Law (IHL), and international stability. IHL is central to this debate, as there is consensus that AWS can be only deployed insofar as they abide by the IHL principles of necessity, proportionality, and distinction. These principles are uncontroversial; what is problematic is understanding whether, and to what extent, autonomous artificial agents enabling AWS can comply with them.^1^ For example, respecting the principle of distinction for AWS is problematic insofar as, at least in its current state of development, autonomous artificial agents are unable to analyse the context in which they operate with the necessary precision to distinguish what/who is a legitimate target (Sharkey, 2010; 2016; Amoroso & Tamburrini 2020).

The IHL principles define ‘operational’ requirements which, if not met by current models of AWS, might be met, at least in theory, in the future by more refined AWS. More fundamental problems emerge when considering AWS and human dignity. In this case the questions is how a person is killed or injured, the focus is on the process through which the decisions to injure or kill are made: if the decision to kill or injure a human being is taken by a machine, then the human dignity of those targeted is violated (Asaro, 2012; Docherty, 2014; Sharkey, 2019; Johnson & Axinn, 2013; Sparrow, 2016; O’Connell, 2014; Ekelhof 2019). The impact of the use of AWS on human dignity is independent from the level of sophistication of the technology, for it questions the legitimacy of delegating the decision on the use of force, possibly lethal force, to machines (Eliav & Benvenisti, 2016). It questions whether delegating this decision is compatible with the values upheld by our societies and refers back to the notions of humanity and public conscience, which are central to legitimacy of any weapons, not only AWS. As the ICRC report stresses.

“ethical decisions by States, and by society at large, have preceded and motivated the development of new international legal constraints in warfare, including constraints on weapons that cause unacceptable harm. In international humanitarian law, notions of humanity and public conscience are drawn from the Martens Clause”, (International Committee of the Red Cross (ICRC), 2018, p. 1).

Ultimately, problems related to human dignity refer to human agency, the decisions and actions that human should and should not delegate, and the moral responsibilities linked to this agency and to the decision to use force. Ascribing moral responsibility for the actions performed by AI systems has proved to be extremely problematic in many domains, the case of AWS is not an exception. As argued by (Taddeo et al., 2021), whilst a responsibility gap is problematic in all the categories of use of AI within the defence and security domain—namely, sustainment and support, adversarial and non-kinetic, and adversarial and kinetic—the gap is particularly worrying when considering the adversarial and kinetic uses of AI, given the high stakes involved (Sparrow, 2007).

Questions also arise with respect to the impact of AWS on international stability. On the one side, AWS may lead to an increased incidence of war and hamper international stability by ‘lowering the barriers’ to warfare (Enemark, 2011; Brunstetter & Braun 2013). For instance, it may be the case that the widespread use of AWS would allow decision-makers to wage wars without the need to overcome the potential objections of military personnel or of a democratic populace more broadly (Steinhoff 2013; Heyns, 2014). In the same vein, asymmetric warfare that would result from one side using AWS may lead to the weaker side resorting to insurgency and terrorist tactics more often (Sharkey, 2012). Because terrorism is generally considered to be a form of unjust warfare (or, worse, an act of indiscriminate murder), deploying AWS may lead to a greater incidence of unjust violence.

Scholarly and policy efforts focusing on these topics have grown over time. However almost ten years later from the DoD executive order and the Human Right Watch report, a shared international (let alone global) approach to address these problems has not yet been defined. The reasons behind this failure are multiple and range from political will, competing interests at the international level, and defence postures, all of which is compounded by a lack of a shared understanding of AWS and of their key features and related ethical and legal implications. As stressed in a UNIDIR report.

“proponents and opponents of AWS will seek to establish a definition that serves their aims and interests. The definitional discussion will not be a value-neutral discussion of facts, but ultimately one driven by political and strategic motivations”, (UNIDIR, 2017, 22).

Indeed, our analysis identified 12 definitions of AWS proposed by States or key international actors—such as the ICRC and NATO. The definitions draw focus on different aspects of AWS and hence lead to different approaches to address the ethical and legal problems of these weapons systems. Clearly, this approach is detrimental both in terms of fostering an understanding of AWS and in facilitating agreement around conditions of deployment and regulations of their use and, indeed, whether AWS are to be used at all. This becomes evident when considering the works of the CCW/GGE. Table 1 below summarises the key points of the discussion of this group between 2014 and 2019. It shows that while there is a consensus on the key aspects of AWS and on the ethical problems that they pose; a shared definition, and therefore a shared understanding, of AWS and of what aspects pose the most pressing ethical and legal problems is still lacking. Consider for example, how the points reported in Table 1 often conflate AWS with LAWS and the related ethical and regulatory problems.

**Table 1:** Key points of the discussions held at the CCW GGE between 2014 and 2019

CCW/GGE	2014	Many interventions stressed the fact that, even if the elaboration of a definition was premature, some key elements appeared as pertinent to describe the concept of autonomy for LAWS, for example the capacity to select and engage a target without human intervention. Some experts highlighted the fact that autonomy should be measurable and should be based on objective criteria such as capacity of perception of the environment, and ability to perform pre-programmed tasks without further human action. Many interventions stressed that the notion of meaningful human control could be useful to address the question of autonomy. Other delegations also stated that this concept requires further study in the context of the CCW. The concept of human involvement in design, testing, reviews, training and use was discussed. The notion of predictability was also underlined by some delegations as a key issue. (Convention on Certain Conventional Weapons, 2014, p. 4)
	2017	The need to improve shared understanding of autonomous weapon systems was recognised. The elaboration of a working definition of LAWS, without prejudice to the definition of systems that may be subject to future regulation, was encouraged. Consideration was given to the scope of a possible definition, including questions of systems already deployed, defensive versus offensive weapons, and the distinction between fully and semi-autonomous systems. The view that it was premature or unhelpful to begin work on definitions was also put forward. (Convention on Certain Conventional Weapons, 2017, p. 7)
	2018	Technical characteristics related to self-learning (without externally-fed training data) and self-evolution (without human design inputs) have to be further studied. Similarly, attempting to define a general threshold level of autonomy based on technical criteria alone could pose difficulty as autonomy is a spectrum, its understanding changes with shifts in the technology frontier, and different functions of a weapons system could have different degrees of autonomy In the context of the CCW, a focus on characteristics related to the human element in the use of force and its interface with machines is necessary in addressing accountability and responsibility. (Convention on Certain Conventional Weapons, 2018, p. 5)
	2019	On the agenda item 5 (b) ‘Characterization of the systems under consideration in order to promote a common understanding on concepts and characteristics relevant to the objectives and purposes of the Convention’ the Group concluded as follows: (a) The role and impacts of autonomous functions in the identification, selection or engagement of a target are among the essential characteristics of weapons systems based on emerging technologies in the area of lethal autonomous weapons systems, which is of core interest to the Group; (b) Identifying and reaching a common understanding among High Contracting Parties on the concepts and characteristics of lethal autonomous weapons systems could aid further consideration of the aspects related to emerging technologies in the area of LAWS.’ (p. 5) ‘(b) Different potential characteristics of emerging technologies in the area of lethal autonomous weapons systems, including: self-adaption; predictability; explainability; reliability; ability to be subject to intervention; ability to redefine or modify objectives or goals or otherwise adapt to the environment; and ability to self-initiate.’ (Convention on Certain Conventional Weapons, 2019, p. 5)

This article aims to fill this gap. We offer a comparative analysis of existing definitions of AWS with the goal of identifying the different approaches that underpin them, their similarities and differences, as well as their limitations. We draw from this analysis to identify essential aspects of AWS and then offer a definition that provides a value-neutral ground to facilitate efforts to address the relevant ethical and legal problems. In doing so, we aim to fill the gap identified by UNIDIR (2017, p. 22). In particular, we identify four key aspects—autonomy; adapting capabilities of AWS; human control; and purpose of use—as the essential factors to define AWS and which are key when analysing the related ethical and legal implications.

Before moving forward with our analysis, we should clarify that, for the purpose of this article, we focus on AWS and consider LAWS as a subset of this category. LAWS are AWS with a specific purpose of use, i.e. deploying lethal force, as opposed to the wider set of purposes of use of AWS, e.g. anti-material, damage, and destruction. In terms of the scope of our analysis, this enables us to consider a wider set of technologies and purposes of use. It should be stressed that ethical problems related to AWS—e.g. issues of control, responsibility, predictability—apply a fortiori when considering LAWS. At the same time, LAWS pose specific ethical problems—e.g. respect of human dignity and of military virtue—related to the lethal purpose of their use.

## Definitions of Autonomous Weapon Systems

We identified 12 definitions of AWS or LAWS (Table, 2) provided by States (either endorsed or retrieved from official documents) and by international organisations, like the ICRC and NATO.^2^ This plethora of definitions encroaches upon international debate on the ethical and legal implications of AWS. For example, it has been reported^3^ that as of August 2020, 30 states declared their endorsement of a pre-emptive AWS ban. However, without a shared understanding of what AWS are, it is hard to identify AWS to ban, let alone enforce any ban of AWS.

China offers a good example of the case in point. Roberts et al. (2020) highlight that Chinese military officials express concerns about the use of AI for kinetic and aggressive purposes and that these concerns motivate the Chinese support to restrict the use of AWS, as expressed at the 5th Convention on CCW and, in the more recent call, supporting the banning of use of LAWS. However, they also stress that “the definition of autonomy embraced by China is extremely narrow, as it focuses only on fully autonomous weapons (Kania, 2018 emphasis added)” (p. 63) and leaves unaddressed AWS that may have lower levels of autonomy.

This is the case with other definitions also focusing on full autonomy. Like the UK definition, which centres on fully autonomous systems “capable of understanding higher-level intent and direction”. The UK is ‘out of step’ for its primary focus on the ‘intention’ of the system, whilst its international partners focus on human (non)intervention with the system (Select Committee on Artificial Intelligence, 2018, p. 105). This point has been further affirmed in various meetings of the GGE and in a report by the House of Lord’s Select Committee on Artificial Intelligence.^4^ The definition refers to cognitive capabilities that AI systems do not possess currently and are very unlikely to gain in the future (Floridi, 2014; Wooldridge, 2020). Indeed, “capable of understanding higher-level intent and direction” defines an atypically high threshold for what is to be considered ‘autonomous’. France’s definition is provided in the same vein, it explicitly mentions that AWS as the ones it defines “do not currently exist”.

Considered from a broader perspective, this approach has the effect of informing future directions of technological innovation by indicating limits to possible uses of AI technologies. In doing so, it may enable regulation to gain an advantage over technological innovation. But this approach rests on a paternalistic view of the role of regulations and regulator, which is problematic per se and may have the undesired effect of hampering technological innovation. When considering AWS specifically, defining the governance of these systems by focusing on futuristic scenarios is detrimental for two reasons. First, focusing on systems that are not currently developed or whose characteristics are technologically unfeasible diverts focus from pressing ethical and legal problems posed by existing AWS and those that may be deployed in the foreseeable future. Second, it undermines regulations and declarations about banning AWS, insofar as these refer to hypothetical AWS with features that current and foreseeable systems do not have, for example ‘understanding’ and ‘intent’. In this case, the implication is that official declaration of banning AWS refers to systems which do not exist yet, and leaves unaddressed other systems, currently being developed. For example, Article36 stressed, that statements made by the UK such as “we have no plans to develop or acquire such weapons,” as reported by its definition (Table 2),

“could appear progressive without actually applying any constraint on the UK’s ability to develop weapons systems with greater and greater autonomy” (Article36, 2018, p. 1).

**Table 2:** Twelve definitions of AWS and LAWS as provided by states or international organisation between 2012 and 2020

State/Organisation	Date	Definition
Canada	2018	“[S]ystems with the capability to independently compose and select among various courses of action to accomplish goals based on its [information] and understanding of the world, itself, and the situation. * * Whilst Canada has no ‘official’ definition, this is the definition used by the Department of National Defence (DND). (Department of National Defence, 2018; see also: Ariel Shapiro, 2019)
China	2018	LAWS should include but not be limited to the following 5 basic characteristics. The first is lethality, which means sufficient pay load (charge) and for means to be lethal. The second is autonomy, which means absence of human intervention and control during the entire process of executing a task. Thirdly, impossibility for termination, meaning that once started there is no way to terminate the device. Fourthly, indiscriminate effect, meaning that the device will execute the task of killing and maiming regardless of conditions, scenarios and targets. Fifthly evolution, meaning that through interaction with the environment the device can learn autonomously, expand its functions and capabilities in a way exceeding human expectations (China, 2018, p. 1) This definition differs from the definition set out by the People’s Liberation Army in 2011: [LAWS are] a weapon that utilizes AI to automatically pursue, distinguish, and destroy enemy targets; often composed of information collection and management systems, knowledge base systems, assistance to decision systems, mission implementation systems, etc. (Kania, 2018)
France	2016	Lethal autonomous weapons are fully autonomous systems. LAWS are future systems: they do not currently exist. […] LAWS should be understood as implying a total absence of human supervision, meaning there is absolutely no link (communication or control) with the military chain of command. […] The delivery platform of a LAWS would be capable of moving, adapting to its land, marine or aerial environments and targeting and firing a lethal effector (bullet, missile, bomb, etc.) without any kind of human intervention or validation. […] LAWS would most likely possess self-learning capabilities (République Française, 2016, pp. 1–2) Additionally: Given the complexity and diversity of environments (particularly in urban areas) and the difficulty of building value-laden algorithms capable of complying with the principles of international humanitarian law (IHL), a LAWS would most likely possess self-learning capabilities, since it seems unrealistic to pre-program all the scenarios of a military operation. This means, for instance, that the delivery system would be capable of selecting a target independently from the criteria that have been predefined during the programming phase, in full compliance with IHL requirements. With our current understanding of future technological capacities, a LAWS would therefore be unpredictable (République Française, 2016, p. 2)
Germany	2020	LAWS [are] weapons systems that completely exclude the human factor from decisions about their employment. Emerging technologies in the area of LAWS need to be conceptually distinguished from LAWS. Whereas emerging technologies such as digitalization, artificial intelligence and autonomy are integral elements of LAWS, they can be employed in full compliance with international law (Federal Foreign Office, 2020, p. 1)
ICRC	2016	Any weapon system with autonomy in its critical functions. That is, a weapon system that can select (i.e. search for or detect, identify, track, select) and attack (i.e. use force against, neutralize, damage or destroy) targets without human intervention. (International Committee of the Red Cross, 2016, p. 1)
Israel	2018	In Israel's view, the shared starting point for this discussion must be that all weapons, including LAWS, are and will always be utilized by humans. We should stay away from imaginary visions where machines develop, create or activate themselves—these should be left for science-fiction movies. As far as terminology is concerned, that means that LAWS should not be regarded as “deciding” anything. Humans are always those who decide, and LAWS are decided upon (Yaron, 2018, p. 2)
NATO		Automated system: a system that, in response to inputs, follows a predetermined set of rules to provide a predictable outcome Autonomous system: a system that decides and acts to accomplish desired goals, within defined parameters, based on acquired knowledge and an evolving situational awareness, following an optimal but potentially unpredictable course of action. (NATO, 2020, p. 16)
Norway	2017	Norway has not yet concluded on a specific legal definition of the term 'fully autonomous weapons systems'. Generally speaking, however, in using the term, we refer to weapons that would search for, identify and attack targets, including human beings, using lethal force without any human operator intervening. These must be distinguished from weapons systems already in use that are highly automatic, but which operate within such tightly constrained spatial and temporal limits that they fall outside the category of 'fully autonomous weapons'. (Norway, 2017, p. 1)
Switzerland		Weapons systems that are capable of carrying out tasks governed by IHL in partial or full replacement of a human in the use of force, notably in the targeting cycle (Switzerland 2016, p. 2)
The Netherlands	2017	A weapon that, without human intervention, selects and engages targets matching certain predefined criteria, following a human decision to deploy the weapon on the understanding that an attack, once launched, cannot be stopped by human intervention. (The Netherlands 2017, p. 1)
United Kingdom^1^	2018	An autonomous system is capable of understanding higher-level intent and direction. From this understanding and its perception of its environment, such a system is able to take appropriate action to bring about a desired state. It is capable of deciding a course of action, from a number of alternatives, without depending on human oversight and control, although these may still be present. Although the overall activity of an autonomous unmanned aircraft will be predictable, individual actions may not (Ministry of Defence, 2018a, p. 13)
	2016	UK CCW GGE contribution UK understands such a system [fully autonomous LAWS] to be one which is capable of understanding, interpreting and applying higher level intent and direction based on a precise understanding and appreciation of what a commander intends to do and perhaps more importantly why […] Critically, this understanding is focused on the overall effect the use of force is to have and the desired situation it aims to bring about. From this understanding, as well as a sophisticated perception of its environment and the context in which it is operating, such a system would decide to take—or abort—appropriate actions to bring about a desired end state, without human oversight, although a human may still be present. The output of such a system could, at times, be unpredictable—it would not merely follow a pattern of rules within defined parameters. (Foreign & Commonwealth Office 2016, p. 2)
US Department of Defence	2012	A weapon system that, once activated, can select and engage targets without further intervention by a human operator. This includes human-supervised autonomous weapon systems that are designed to allow human operators to override operation of the weapon system, but can select and engage targets without further human input after activation. (US Department of Defense, 2012, p. 13–14)

^1^The UK adopted the NATO definition of autonomous systems, but it did not abandon any of its previous definitions provided in 2016 and 2018. This is problematic insofar as UK definitions and the NATO definition set different requirements to identify AWS, which taken together may hamper attempts to define national, coherent, policy approaches to AWS.

Indeed, the high threshold established by the UK to identify AWS will, if unchanged, permit the UK ever-increasing use of AWS insofar as these do not show “understanding higher-level intent and direction”. The problem in this case is conceptual: the restrictive definition of AWS does not enable the correct categorization of these systems, which are autonomous, but that do not meet the high threshold posed by the UK definition. These systems either fall into a grey area between both categories or are mistakenly lumped into the more familiar ‘automatic’, missing the opportunities to consider and address the ethical and legal problems that they pose.

To avoid these limitations, it is important to define AWS by focusing on their characterising aspects –e.g. autonomy—and describe them following the understanding that scientific and technological research have of them. In this way, the definition can offer a rigorous tool to identify AWS and avoid the inclusion of unsubstantiated characteristics of these systems. The goal of the definition, as the ICRC states, is that it.

“encompasses some existing weapon systems, [and so] enables real-world consideration of weapons technology to assess what may make certain existing weapon systems acceptable—legally and ethically—and which emerging technology developments may raise concerns under international humanitarian law (IHL) and under the principles of humanity and the dictates of the public conscience” (International Committee of the Red Cross, 2016, p. 1)

This is for example the driving rational of the ICRC definition (see Table 2), and the outcome of the US definition, which considers autonomy on a function-based spectrum vis-à-vis human engagement so it can also encompass *existing* weapons systems (International Committee of the Red Cross, 2016, p. 1; US Department of Defense, 2012, pp. 13–14). While being inclusive, however, it is also important to maintain some level of specificity to avoid too generic an approach that may then generate confusion in identifying AWS. This is the risk linked to the NATO definition (see Table 2). It is true that the definition is not meant to focus specifically on AWS but on autonomous systems in general, but it is too generic even for this purpose. For example, it refers to “desired goals” leaving unspecified whether these are the political, organisational, strategic or tactic goals or the specific goals that a system may have or acquire. Similarly, it refers to “situational awareness”, but it is unclear whether this is meant to be an understanding of the immediate context of deployment of the system or of the wider strategic scenario.

From the analysis of the definitions reported in Table 2, four characteristics can be extracted as recurring more often in the reported definitions, namely: autonomy, adapting capabilities, human intervention and control, and purpose of use. While these characteristics point in the right direction when considering what AWS are, for example, they resonate with the definition of AI adopted in (Taddeo, 2019; Taddeo et al., 2021) of a form of autonomous, self-learning agency; the way in which they are described is, at times, conceptually misleading. The next three subsections analyse these characteristics to clarify their implications with respect to the ethical and legal debate on AWS.

### Autonomy, Intervention, and Control

Autonomy is a central element of all the definitions of AWS. In some cases, it is assumed to mean the ability of a system to operate successfully without human intervention. The German definition, for example, mentions machines that “completely exclude” humans from the decision-making process. In other cases, autonomy is conflated with the lack of human control. This is the case of the French definition, for instance, which qualifies human intervention as.

“human supervision, meaning there is absolutely no link (communication or control) with the military chain of command”. (République Française, 2016, p. 1)

As we will see in Sect. 3.1., this assumption is misleading both conceptually and operationally. An artificial system can be, in principle, fully autonomous, insofar as it can operate independently from a human or of another artificial agent, and yet be deployed under some form of meaningful human control.

The distinction between autonomy and control is important for three reasons. First, conceptual clarity: it avoids considering automation and human control as mutually exclusive concepts: automation makes human intervention unnecessary but does not make human control impossible. This is why the DoDD 3000.09 is correct in referring explicitly to ‘human-supervised autonomous weapons systems’^5^ and to distinguish them from ‘semi-autonomous weapon systems’, whose autonomy is circumscribed to “engagement related functions” but depend on a human operator for the target selection.

Distinguishing autonomy from control brings a second and a third advantage, as it future-proofs the debate on AWS. Many of the problems posed by AWS do not concern the desirable level of autonomy of these systems, but the desirable level of control over these systems. The decision about control is in many ways normative, insofar as it is not only defined by the technological affordances (i.e. how much autonomy a system can have) but also, and more importantly, by the decisions and tasks that should be delegated to machines without envisaging human control. Separating the two concepts, enables a focus on normatively desirable forms of control irrespectively of the level of autonomy that these machines may acquire someday.

The third advantage of this distinction, is that it pre-empts approaches that leverage the lack of existing examples of fully autonomous AWS to avoid discussing their regulation as claimed, for example, by the Russian Federation.

“Certainly, there are precedents of reaching international agreements that establish a preventive ban on prospective types of weapons. However, this can hardly be considered as an argument for taking preventive prohibitive or restrictive measures against LAWS being a by far more complex and wide class of weapons of which the current understanding of humankind is rather approximate”, (Russian Federation, 2017, p. 2)

### Adapting Capabilities

Of the 12 definitions considered in this review, only the French and the Chinese definitions stress the adapting capabilities, specifically the definitions mention learning capabilities of AWS as a key characteristic. The lack of focus on adapting capabilities in general in the definition of AWS is problematic, as these are a key feature of AI technologies, which increasingly underpin AWS.

AWS can function without adapting capabilities. For example, they may rely on rule-based programming^6^ which enable an autonomous reaction to environmental triggers but do not allow for planning different behaviours when the environment changes. One can imagine a sensor detecting an incoming object and the algorithm triggering a response of the system, e.g. fire to destroy the object.

However, systems based on rule-based algorithms are increasingly being replaced by AI-based system. Military institutions are investing in AI for a wide range of applications, for example significant efforts are already underway to harness developments in image, facial and behaviour recognition using AI and machine learning techniques for intelligence gathering and “automatic target recognition” to identify people, objects or patterns.^7^

Disregarding adapting capabilities in the definitions of AWS leads to disregarding key characteristic of these systems and hinders the debate on their ethical and legal implications. Crucially, these capabilities pose questions with respect to the predictability, and hence the trustworthiness, of these systems (Taddeo, 2010; Taddeo, 2017; Taddeo et al., 2019) and with respect to the attribution of responsibilities of the actions that these systems perform as well as with the implementation of meaningful forms of control.

The French definition stresses that learning capabilities would be necessary to adapt to the complexity of operation scenarios which cannot be foreseen and thus “pre-programmed” in the system. It also stresses that this means.

“that the delivery system would be capable of selecting a target independently from the criteria that have been predefined during the programming phase, in full compliance with IHL requirements. With our current understanding of future technological capacities, a LAWS would therefore be *unpredictable*”. (emphasis added, République Française, 2016, p. 2)

A similar point is also highlighted in (International Committee of the Red Cross (ICRC), 2018),

“the application of AI and machine learning to targeting functions raises fundamental questions of inherent unpredictability” (p. 2).

Learning capabilities, and the related unpredictability of outcomes, also pose problems with respect to Article 36 of Additional Protocol I to the Geneva Conventions on weapons review.

As reported in UNIDIR (2017):

“From a technical perspective, any system that continues to learn while deployed is constantly changing. It is not the same system it was when deployed or verified for deployment. Some have raised questions about the legality of adaptive systems, particularly in regards to States’ Article 36 obligations”, (p. 10).

This is crucial, as remarked by ICRC.

The ability to carry out [an Article 36] review entails fully understanding the weapon’s capabilities and foreseeing its effects, notably through testing. Yet foreseeing such effects may become increasingly difficult if autonomous weapon systems were to become more complex or to be given more freedom of action in their operations, and therefore become less predictable (as reported in UNIDIR, 2017, p. 26) .

For both ethical and legal reasons, hence, the focus on adapting capabilities of AWS is essential. It is the nature of the adapting process which raises both significant opportunities and challenges and sets AI-enabled systems apart from highly automated rules-based systems. Adapting capabilities qualify the latest and future generations of AWS. Focusing on them allows for further clarification of the distinction between automatic and autonomous systems (more on this in Sect. 3); and for identifying the source of a number of key ethical and legal implications of AWS. This is why, it is important that definitions of AWS mention these capabilities expressly, and it is problematic that even the two most comprehensive definitions—the US and the ICRC—of AWS fail to grasp this point, missing the opportunity to cast light on a key element of these systems.

### Purpose of Deployment

Most of the definitions qualify the purpose of deployment implicitly, by reference to ‘weapons’ and by the fact that AWS are deployed in kinetic contexts. These two elements indicate some form of destructive (whether anti-material or lethal) use of these systems. However, it is important to understand the range of possible uses with greater precision, for example considering the specific tasks that AWS may undertake within the context of kinetic operations.

Of the definitions reported in Table 2, four (Canada, Israel, Germany, and UK) do not mention explicitly any specific purpose of deployment. The kinetic outcome of the use of AWS is somehow assumed in this case, leaving undefined for example whether AWS will be used for deliberate or dynamic targeting. Of the other eight definitions, one (NATO) does not mention any specific purpose (it should be stressed, however, that NATO definition is of autonomous systems in general and not of AWS), the remaining definitions refer to the purposes of use of AWS as to deploy lethal force (China and France) or more specifically to select and engage targets (whether non-humans or humans) to be neutralised, damaged or destroyed (ICRC, Norway, Switzerland, The Netherlands, US).

All the definitions leave unaddressed the specific steps of the tasks that are delegated to machines. These steps, however, are key when considering AWS. Consider for example criticisms posed by (Roff, 2014) to the US definition, Roff stresses that the meaning of ‘select’ in ‘select and engage’ is unclear, insofar it is not clear whether this also includes the detection of targets.^8^ As she clarifies, if detection is not included, then we may assume that it is carried out by a human, thereby obviating important ethical (and technical) questions.

Roff’s criticism highlights the complexity of these tasks and of the processes underpinning the decision to deploy force. Consider for example the steps underpinning targeting decision as described in (Ekelhof & Persi Paoli 2021). They outline a complex process, which extends across the decision and command chain when considering AWS. The process includes tasks and decisions spanning the tactical, operational, strategic and political levels, which are often interlinked. The complexity of the process requires a more specific approach when considering the tasks performed by AWS. This is achieved in two ways, by specifying explicitly the purposes of deployment—at a high Level of Abstraction (LoA) —indicating the destructive, whether lethal or not, goal for using these systems; and—at a lower LoA—by specifying which steps in the process of exerting force may be within the remit of the AWS and under which level of human control AWS may operate. The outcome of the ethical and legal analyses of AWS depends on these specifications.

## A Definition of AWS

We offer a value-neutral definition of AWS. In doing so we have a twin-goal of (i) defining the key characteristics that permit the identification of AWS; and (ii) specifying these characteristics so to clarify their relations—e.g. automation vs control, and their differences—e.g. automatic vs autonomous. To do so, we consider autonomy, adapting capabilities, and control as characteristics that can each be mapped on a continuum. AWS can have each of these characteristics to a greater or lower level. We are also inclusive with respect to the set of possible purposes of deployment, with the aim of clarifying what the range may be. Identifying the combination of the different levels and purposes, if any, that meet ethical and legal requirements is the tasks of ethical analyses, of policies and laws, this is why we leave this to the next step of our work. With this approach in mind, we define AWS as follows:

**Definition**: an artificial agent which, at the very minimum, is able to change its own internal states to achieve a given goal, or set of goals, within its dynamic operating environment and without the direct intervention of another agent and may also be endowed with some abilities for changing its own transition rules without the intervention of another agent, and which is deployed with the purpose of exerting kinetic force against a physical entity (whether an object or a human being) and to this end is able to identify, select or attack the target without the intervention of another agent is an AWS. Once deployed, AWS can be operated with or without some forms of human control (in, on or out the loop).

A lethal AWS is specific subset of an AWS with the goal of exerting kinetic force against human beings.

The next subsections will unpack this definition by focusing on the concepts of autonomy, adapting capabilities, and control. The purposes of deployment are less conceptually problematic and thus we will not delve into it. It is important, however, to remark here that the purpose of deployment have been identified as being those directly related to the goal to achieve, i.e. exerting force (Taddeo et al., 2021). Selecting targets and engaging (whether deliberate or dynamic) are directly linked to the purpose of deploying force. Hence, a system whose selecting and attacking functions are autonomous, but which is directed by another agent(s) for all its other purpose of uses, e.g. mobility, would still be considered an AWS.

### Autonomous, Self-Learning, Weapons Systems

A key question underpinning the definition of AWS is the distinction among ‘automatic’, ‘automated’, and ‘autonomous’ systems. Especially the distinction between ‘automated’ and ‘autonomous’ can prove to be difficult when considered from an ethical or a legal LoA. An ICRC report, for example, stresses that.

“There is no clear technical distinction between automated and autonomous systems, nor is there universal agreement on the meaning of these terms […]”, (International Committee of the Red Cross, 2019, p. 7).

In a similar vein, the joint concept note 1/18 on ‘Human–Machine Teaming’ published by the UK Ministry of Defence in 2018 started by remarking that.

“There is no clear, definable and universally agreed boundary between what constitutes automation and what is autonomous,” it states, “because the assessment of autonomy and the term's use is subjective and contextual”, (Ministry of Defence, 2018b, p. 57).

While one may agree that the distinction between automation and autonomy is blurred, this is not because the assessment of autonomy of artificial agents is subjective or context-dependent. Within the field of computer science, and particularly of Agent Theory (Wooldridge & Jennings, 1995; Castelfranchi & Falcone, 2003), there is quite a clear understanding of the differences between these concepts.

Let us consider ‘automatic’ agents first. These are agents whose actions are predetermined and will not change unless acted upon by pre-selected triggers and/or human intervention. Automatic agents are not teleological, they do not pursue a goal, but simply react to an external trigger. In this sense, they are ‘causal entities’ (Castelfranchi & Falcone, 2003). A landmine falls squarely in this category, for its action is causally determined by a specific trigger, such as someone stepping on it. AWS do not belong to this category insofar as their behaviour is not simply reactive to (caused by) the environment.

AWS execute tasks to achieve goals (teleological agents), they can adjust their actions on the basis of the feedback that they receive from the environment (automated artificial agents), may also be able define plans (heuristic artificial agents) to achieve their goals, and may be able to refine their behaviour in response to the changes in the environment (adapting artificial agent). At this point, we can consider AWS as systems that at the very least are automated, teleological artificial agents, but we can be more specific and go a step further.

For the purposes of the definition, it is important to consider what the minimum requirements are for an artificial agent to be autonomous. To do so we will refer to the definitions of autonomous artificial agent provided Castelfranchi’s and Falcone’s (Castelfranchi & Falcone, 2003) and Floridi’s and Sanders’ (2004). The two definitions are given at different LoAs, the reader may consider one (Floridi’s & Senders’) a specification of the other (Castelfranchi’s & Falcone’s).

According to Castelfranchi and Falcone, autonomous agents enjoy the following properties:

“[…] *their behaviour* is *teleonomic*: it tends to certain specific results due to internal constraints or representations, produced by design, evolution, or learning, […];

[…] they do not simply receive an input—not simply a force (energy) but information—but *they* (*actively) “perceive” and interpret their environment* and the effects of their actions;

[…] *they orient themself towards the input*; in other words, they define and select the environmental stimuli;

[…] they have “internal states” with their own exogenous and endogenous evolution principles, and their behaviour also depends on such internal states” (Castelfranchi & Falcone, 2003, p. 105).

Internal states of an artificial agent can be described as the configuration of the agent (for example the layers, the nodes, the value and the weights of a neural network at a specific moment in time) when it is performing a given operation. Internal states are key in the definition of autonomy insofar as the transition from state0 to state1 corresponds to a change of behaviour of the system. How the transition is determined defines the difference between automated and autonomous systems. Indeed, internal states are also key to the definition offered by Floridi and Sanders. Accordingly, an autonomous artificial agent enjoys three characteristics.

“*Interactivity* means that the agent and its environment (can) act upon each other. Typical examples include input or output of a value, or simultaneous engagement of an action by both agent and patient—for example gravitational force between bodies.

*Autonomy* means that the agent is able to change state without direct response to interaction: it can perform internal transitions to change its state. […]

*Adaptability* means that the agent’s interactions (can) change the transition rules by which it changes state. This property ensures that an agent might be viewed, at the given LoA, as learning its own mode of operation in a way which depends critically on its experience […]” (Floridi & Sanders, 2004, 357).

The ability of an artificial agent to change its internal states without the direct intervention of another agent marks (binarily) the line between automatic/automated and autonomous. A rule-based artificial system and a learning one both qualify as autonomous following this criterion.

As mentioned in Sect. 2.1, adaptability is becoming a characteristic increasingly more common for AWS. It is the characteristic that underpins both their potential for dealing with complex, fast-pacing scenarios and the one that leads to unpredictability, lack of transparency, of control, and responsibility gaps related to the use of these agents. Thus, it is important to include adaptability capabilities in the definition of AWS and to offer a clear—to some extent technical—specification of these capabilities to help avoiding anthropomorphising these agents and set a clear, binary, threshold below which one can say that an agent has no adaptability capabilities. This is why in the definition that we propose in this report we refer to an artificial agent endowed with some abilities for changing its transition rules to perform successfully in a changing environment.

### Human Control

The definition provided in Sect. 3 refers to human control as a mode of deploying AWS and not as one of their defining characteristics. This is because the autonomy of AWS is not defined with respect to human control but with respect to the intervention of another agent on the AWS. There are different forms of control, for example Amoroso and Tamburrini (Amoroso and Tamburrini 2020) identify three:

“First, the obligation to comply with IHL entails that human control must play the role of a fail-safe actor, contributing to prevent a malfunctioning of the weapon from resulting in a direct attack against the civilian population or in excessive collateral damages. Second, in order to avoid accountability gaps, human control is required to function as accountability attractor, i.e., to secure the legal conditions for responsibility ascription in case a weapon follows a course of action that is in breach of international law. Third and finally, from the principle of human dignity respect, it follows that human control should operate as a moral agency enactor, by ensuring that decisions affecting the life, physical integrity, and property of people (including combatants) involved in armed conflicts are not taken by non-moral artificial agents ”, (p. 189).

One may disagree with this taxonomy or consider control better defined at a different LoA, for example focusing only on the technical specifications of AWS. However, the relevant literature converges on considering control of AWS as dynamic, multidimensional and situation dependent and as something that can be exercised focusing on different aspects of the human–machine team. For example, the Stockholm International Peace Research Institute and the ICRC identify three main aspects of human control of weapon systems: the weapon system’s parameters of use, the environment, and human–machine interaction (Boulanin et al., 2020). More aspects can also be considered. Boardman and Butcher (2019) suggest that control should not just be meaningful but ‘appropriate’, insofar as it should be exercised in such a way to ensure that the human involvement in the decision-making process remains significant without impairing system performance.

The discussion about what constitute meaningful human control of AWS and whether this can be exerted in an appropriate way does not fall within the scope of this report, as our goal here is to identify the key characteristics of AWS more than the normative conditions for their design, development and deployment. However, to the extent to which our analysis sheds light on these characteristics and their relation, it is important to stress that human control is not antithetical to the autonomy of AWS and can be exerted over AWS at different levels, from the political and strategic decisions to deploy AWS to the kind of tasks delegated to them. The question is which form of control is ethically desirable and should, ideally, be considered by decision- and policy-makers in designing the governance of AWS.

## Conclusion

The debate on AWS is shaped by strategic, political, and ethical considerations. Competing interests and values contribute to polarize the debate, while politically loaded definitions of AWS undermine efforts to identify legitimate uses and to define relevant regulations. These efforts are hindered even further when conceptual confusion is added to this picture. In a famous article laying down the foundation of computer ethics as an area of research Moor (Moor, 1985) wrote:

“A difficulty is that along with a policy vacuum there is often a conceptual vacuum. Although a problem in computer ethics may seem clear initially, a little reflection reveals a conceptual muddle. What is needed in such cases is an analysis which provides a coherent conceptual framework within which to formulate a policy for action” (p. 266).

In this article, we do not provide an ethical framework to assess the mora permissibility of AWS. Here, we aim to overcome the conceptual muddle around AWS. We do so in two ways: the comparative analysis and the value-neutral definition. The comparative analysis of the official definitions helps in identifying key points of conceptual confusions, e.g. the distinction between automatic and autonomous or the one between autonomy and control. It also highlights a serious gap in these definitions, as to the reference to adapting capabilities of these systems.

The value-neutral definition is not informed by policy or strategic aims, nor does it include normative aspects. It has been designed considering key technical characteristics of these systems and with the sole purpose of enabling the identification of AWS and to distinguish these systems from other weapon systems, like automatic ones. Irrespective of the next steps in our research, we believe that having a value-neutral definition of AWS will help academic and policy debates on this topic, as it offers a shared ground on which different views can be confronted.

## Footnotes

1 See (Blanchard & Taddeo, 2022a, b, c; Taddeo & Blanchard, 2022) forthcoming articles on ascribing moral responsibility for the actions of AWS, and for the application of the principles of distinction, proportionality, and necessity to AWS.

2 NATO offers a definition of *autonomous systems* and not specifically of AWS. Nonetheless, we include it here insofar as it refers to identifying characteristics of AWS.

3 https://www.hrw.org/report/2020/08/10/stopping-killer-robots/country-positions-banning-fully-autonomous-weapons-and

4 (Select Committee on Artificial Intelligence, 2018, 105) Nb. On 24th April 2019 Lord Browne tabled a question in the HoL asking what representations the Government had received from the MoD regarding the recommended that the UK align its definition of AWS with that of international partners. The Government noted that it had received some representations but nevertheless pointed to the fact that “the UN Convention on Certain Conventional Weapons Group of Government Experts on Lethal Autonomous Weapons Systems is yet to achieve consensus on an internationally accepted definition or set of characteristics for autonomous weapons.” (House of Lords, 2019).

5 (Department of Defense 2012, 14).

6 Rule-based systems are artificial systems showing autonomous responses to an input, however these systems operate following predetermined rules and are not able to change these rules, and hence their behaviour, to adapt to the environment in which they act.

7 See for example, https://www.sbir.gov/sbirsearch/detail/1413823; https://www.icrc.org/en/document/artificial-intelligence-and-machine-learning-armed-conflict-human-centred-approach; and https://blogs.icrc.org/law-and-policy/2019/03/19/expert-views-frontiers-artificial-intelligence-conflict/

8 (Ariel, 2016).
